# Optimal timing and cutoff range of lung ultrasound in predicting surfactant administration in neonates: A meta-analysis and systematic review

**DOI:** 10.1371/journal.pone.0287758

**Published:** 2023-07-27

**Authors:** Keren Luo, Haoran Wang, Fangjun Huang, Jun Tang

**Affiliations:** 1 Department of Neonatology, West China Second Hospital, Sichuan University/Key Laboratory of Birth Defects and Related Diseases of Women and Children (Sichuan University), Ministry of Education, Chengdu City, Sichuan Province, China; 2 West China School of Medicine, Sichuan University, Chengdu City, Sichuan Province, China; St Paul’s Hospital Millennium Medical College, ETHIOPIA

## Abstract

**Objective:**

Timely application of surfactant replacement therapy is critical for neonates with respiratory distress syndrome (RDS). Presently, early clinical decision on surfactant use relies solely on ventilator parameters. However, ventilator parameters are unable to truly recapitulate the extent of surfactant deficiency. Lung ultrasound has been increasingly used in the early prediction of surfactant use in recent years, but its predictive value remains unclear. Therefore, we conducted this study to examine its predictive value in surfactant use and determine the optimal timing and cutoff value.

**Methods:**

Studies on neonates with respiratory distress or diagnosed with RDS were collected from PubMed, Embase, Cochrane Library, and Web of Science. Primary outcomes included sensitivity, specificity, and positive and negative predictive values of lung ultrasound.

**Results:**

Ten eligible studies with 1162 participants were included. The sensitivity and specificity of lung ultrasound in predicting surfactant use were 0.86 (95% CI: 0.81–0.90) and 0.82 (95% CI: 0.71–0.90), respectively. Lung ultrasound performed within 1–3 h after birth had a sensitivity of 0.89 (95% CI: 0.79–0.95) and a Youden’s index of 0.67. Compared with a lung ultrasound score (LUS) cutoff of ≤6/7, ≤8, >5, >6/7, and >8, a LUS cutoff of ≤5 had higher Youden’s index (0.73) and sensitivity (0.94, 95% CI: 0.85–0.97) in predicting surfactant use.

**Conclusions:**

Lung ultrasound is effective for predicting surfactant use in neonates. Lung ultrasound within 1–3 h after birth and a LUS cutoff of 5 are recommended. However, the symptoms and oxygenation of the neonatal patients must also be considered.

## Introduction

Surfactant replacement therapy has been shown to effectively improve early respiratory distress and late prognosis of respiratory distress syndrome (RDS) neonates within 3 h of birth [1]. Current clinical practices emphasize the early use of surfactant to improve prognosis of neonates exhibiting symptoms of respiratory distress, such as shortness of breath, grunting, dyspnea, and transcutaneous oxygen saturation of <0.90 in the absence of oxygen inhalation [2]. However, not all neonates require surfactant since those with transient tachypnea of the newborn (TTN) may exhibit these symptoms. Therefore, clinical guidelines [3] and expert consensus [4] have added the use of ventilator parameters when determining surfactant use. The 2019 European Consensus Guidelines recommended FiO2 > 0.30 as the dosing cutoff for all neonates with clinically diagnosed RDS [3]. In addition, the 2021 Chinese Expert Consensus specified that a CPAP ≥6 cmH2O is an indication for surfactant use [4]. However, ventilator parameters are unable to truly recapitulate the extent of surfactant deficiency and are largely influenced by the type of respiratory support and degree of oxygenation. Thus, there are limitations in solely relying on clinical symptoms and ventilator parameters when determining surfactant use, which can result in unnecessary surfactant use or missing the best timing of surfactant.

Due to its timeliness, convenience, and radiation-free advantages, lung ultrasound has gained wide application in the neonatal ward in recent years. Two meta-analyses in 2020 pointed out that lung ultrasound has relatively high sensitivity and specificity for diagnosing neonatal RDS and may replace chest X-ray as the diagnostic measure [5, 6]. In addition, lung ultrasound has also demonstrated relatively high accuracy in diagnosing neonatal TTN [7]. Furthermore, Liu et al. [8] established standards and guidelines for the instrument operation and parameter setting of lung ultrasound in neonates, which further improved the accuracy and reliability of the procedure. Though, it is worth noting that qualitative evaluation by lung ultrasound is somewhat subjective, which renders the procedure difficult for wide clinical application. Moreover, only using lung ultrasound as a means of disease diagnosis cannot maximize its role in assessing pulmonary oxygenation in neonates. Consequently, Brat et al. [9] designed a scoring system that allows semi-quantitation by lung ultrasound. Furthermore, other studies have used the distribution of A-lines and B-lines to classify lung ultrasound images into different types [10, 11]. It has been reported that lung ultrasound score (LUS) can effectively identify failure of noninvasive assisted ventilation in neonatal RDS patients [11–14]. This then raises the question of whether LUS can predict surfactant use in RDS patients. Although many studies have confirmed the significance of LUS in guiding surfactant use [9, 15–26], the LUS cutoff value was inconsistently selected based on gestational age [9] or different timing of lung ultrasound [22]. Here, we performed a meta-analysis of the predictive value of lung ultrasound in surfactant use and assessed the optimal LUS cutoff value and timing of lung ultrasound to standardize the clinical application of surfactant and to rationalize and refine the treatment of neonatal diseases.

## Methods

This systematic review and meta-analysis was conducted in accordance with the PRISMA statement [27] and has been registered on PROSPERO (Registration No. CRD42022359004) [28].

### Inclusion and exclusion criteria

The included studies were diagnostic accuracy studies, prospective or retrospective cohort and case-control studies. Neonates with signs of respiratory distress or diagnosed with RDS were included. Neonates with chromosomal abnormalities, congenital malformations, congenital lung diseases, and meconium aspiration syndrome, as well as those requiring delivery room surfactant administration were excluded from the study.

### Literature search

PubMed, Embase, Cochrane Library, and Web of Science were searched for relevant articles from inception to September 8th, 2022, with no language restriction. Detailed search strategy is provided in S1 File.

### Literature screening and data extraction

Literature screening and data extraction were performed by two independent reviewers (Luo and Wang), and any disagreements were consulted with and settled by a third reviewer (Huang).

Data including title, first author, year of publication, study type, study background, number of patients receiving surfactant treatment, total number of patients, standards for surfactant use, timing of lung ultrasound, lung ultrasound evaluation criteria, LUS cutoff, sensitivity, and specificity were extracted into a pre-designed form.

### Outcome measures

The outcome measures included sensitivity, specificity, positive and negative predictive values of lung ultrasound, optimal LUS cutoff, and optimal timing of lung ultrasound.

### Quality assessment

The methodological qualities of the eligible articles were assessed using the Quality Assessment of Diagnostic Accuracy Studies 2 (QUADAS-2) scoring system. Two authors (Luo and Wang) independently assessed the methodological quality of the included studies and extracted data using RevManager 5.4. Discrepancies were consulted with and settled by a third reviewer (Huang).

### Data compilation and analysis

Data were fitted to the bivariate mixed effect model using the "midas" command of Stata 15.0 (StataCorp LLC, College Station, TX), and the point estimates of test sensitivity and specificity, positive likelihood ratio, negative likelihood ratio, diagnostic odds ratio, summary receiver operating characteristic (SROC) curve, Youden’s index, and the corresponding 95% confidence intervals (Cis) were calculated. Comprehensive SROC curves were plotted, and the area under the curve (AUC) and its 95% CI were calculated. Publication bias was determined by Deeks’ funnel plot, and heterogeneity among studies was determined by Q statistics and I^2^ statistics. An I^2^ >50% indicates high heterogeneity, and a P<0.05 was considered statistically significant. Subgroup analyses were performed to discuss sources of heterogeneity. Subgroup analysis was performed on the timing and LUS cutoff of lung ultrasound. To ensure the reliability of the conclusion, each subgroup contained at least 4 sets of data. Different cutoff values were used for meta-analysis, and the optimal cutoff values were selected according to the maximum Youden index.

## Results

### Study selection

**Fig 1:**
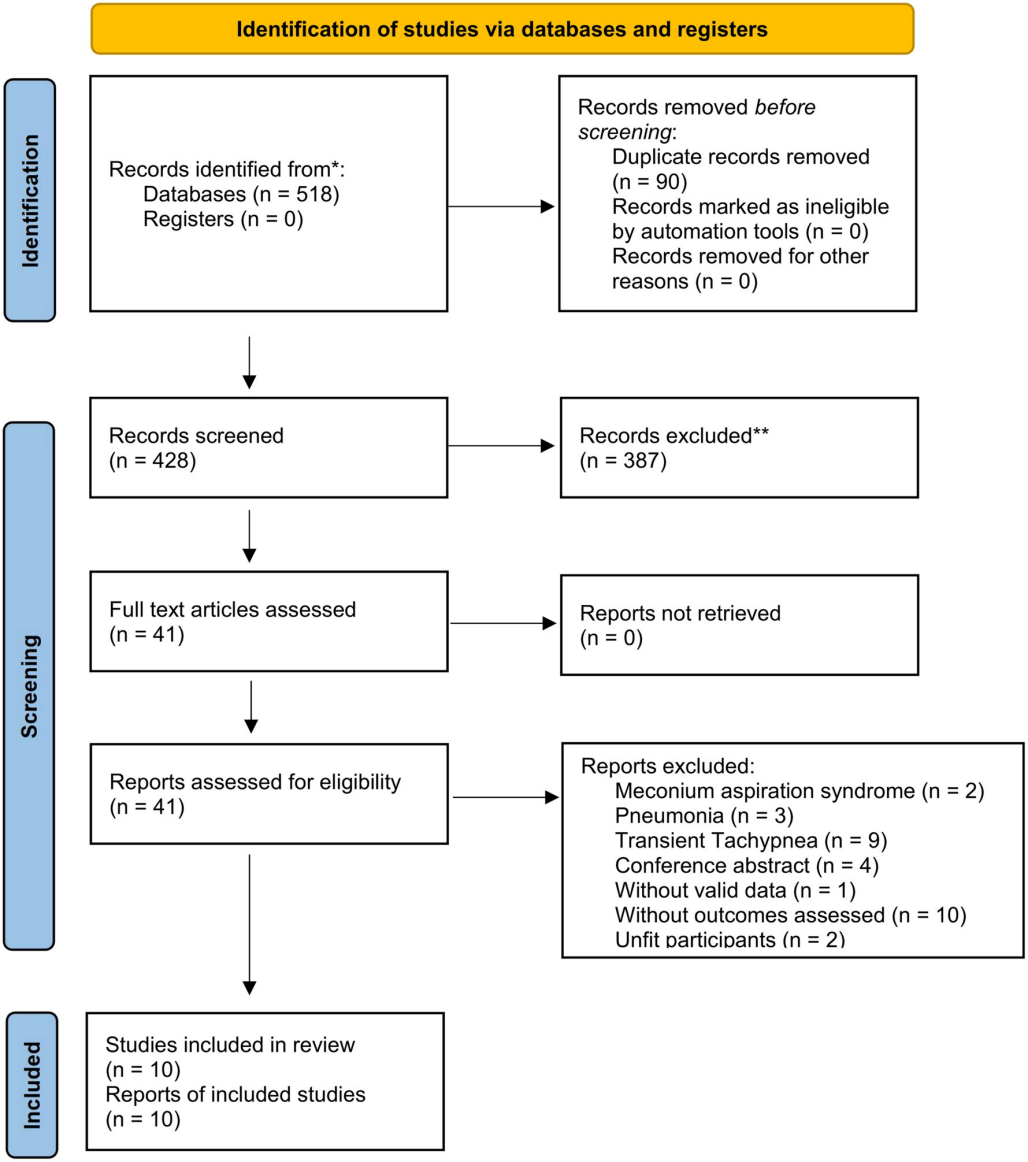
Article screening workflow.

After reading the titles, abstracts and full texts of the 518 related articles, a total of 10 eligible studies with 1162 participants were included.[9, 15, 17–20, 22–24, 26] The flow diagram for study selection is shown in Fig 1. Some of the studies that were excluded for varying reasons are summarized in S1 Table.

### Study characteristics

Among the included studies, 5 studies involved neonates diagnosed with RDS [15, 17, 23, 24, 26] and 5 studies involved neonates with symptoms of respiratory distress [9, 18–20, 22]. Five of the studies clearly stated that subjects were first provided with noninvasive ventilation or CPAP [15, 17–20]. With regards to the standards for surfactant use, 7 studies [9, 17, 19, 20, 22, 23, 26] referenced the 2013 [29], 2016 [30], and 2019 [3] European Consensus Guidelines and emphasized on FiO2 monitoring. The FiO2 cutoff was set at 0.3 and 0.4 for extremely and very preterm infants in both the 2013 01:39 PM and 2016 [30] guidelines, respectively, and at 0.3 in the 2019 [3] guidelines. The other 3 articles [15, 18, 24] did not clearly state which guidelines were used for designing the standards for surfactant use but still highlighted the need to focus on ventilator parameters such as FiO2 and SpO2. With regards to the diagnostic criteria for lung ultrasound, 9 articles [9, 15, 17, 18, 20, 22–24, 26] employed the 3-region 18-point scoring system established by Brat et al. [9] in evaluating the lung ultrasound results. One article [19] performed qualitative evaluation by classifying lung ultrasound images into different types based on A-line and B-line distribution [11] but did not provide the diagnostic data. For study type, 9 of the 10 studies were cohort studies [9, 15, 17–20, 22, 23, 26]. Among these studies, those conducted by Brat et al. [9] and Perri et al. [22] set multiple LUS cutoffs based on gestational age or timing of lung ultrasound and obtained the corresponding diagnostic results. On the other hand, De Martino et al. [17] calculated sensitivity and specificity using 3 different LUS cutoffs. One study [24] was a nested cohort study, which concluded that lung ultrasound is helpful for the timely application of surfactant and for improving RDS prognosis. However, this study did not provide diagnostic results and was hence not included in the final data analysis. The basic characteristics of the included studies are shown in S2 Table.

### Risk of bias in studies

**Fig 2:**
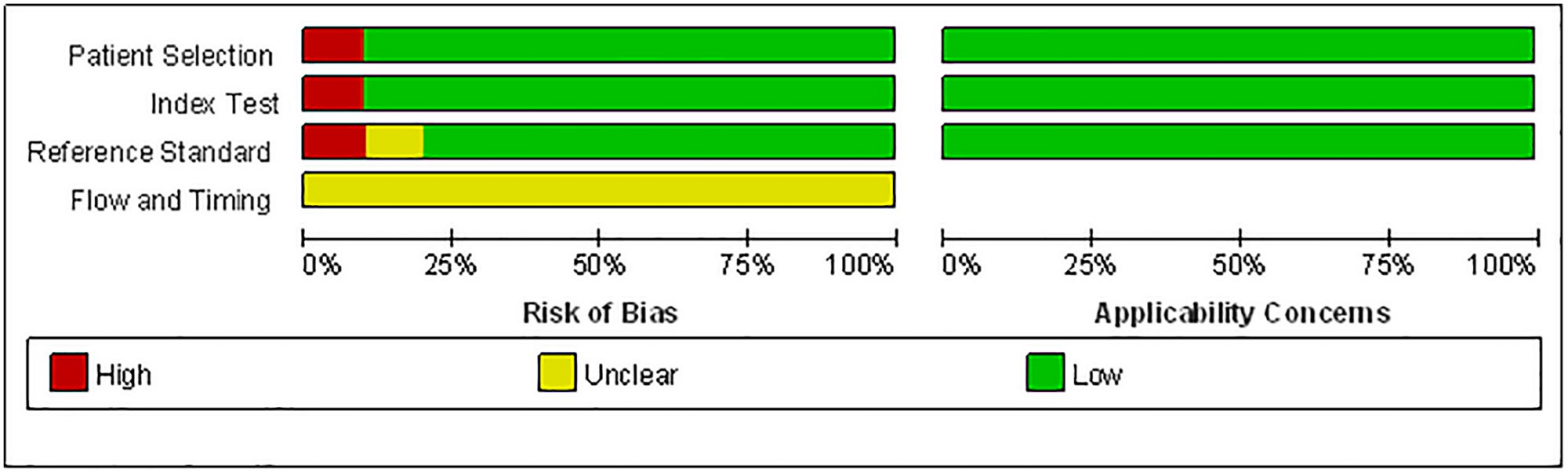
Risk of bias assessment of the included studies.

We evaluated the included studies using the QUADAS-2 tool. All studies avoided a case-control design and a pre-specified threshold, as well as interpreted the reference standard results without knowledge of the results of the index tests, except for a nested cohort study [24], which used a pre-specified cutoff for evaluating lung ultrasound. All the studies could not determine there was an appropriate interval between index test and reference standard (Fig 2).

### Surfactant therapy as primary outcome

**Fig 3:**
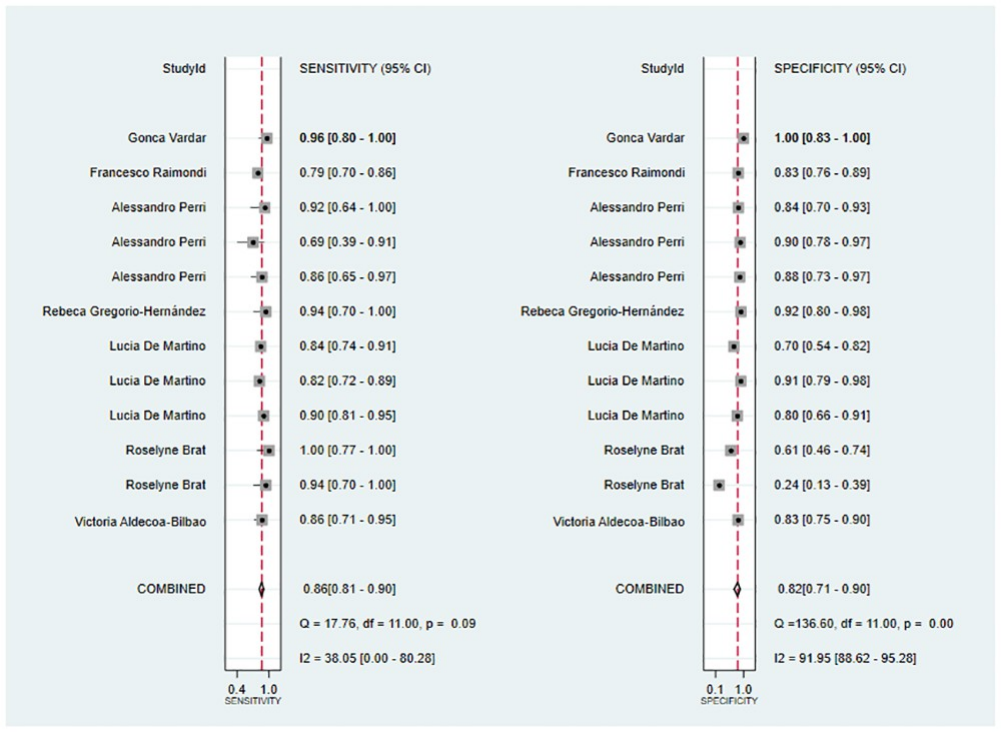
Forest plot.

**Fig 4:**
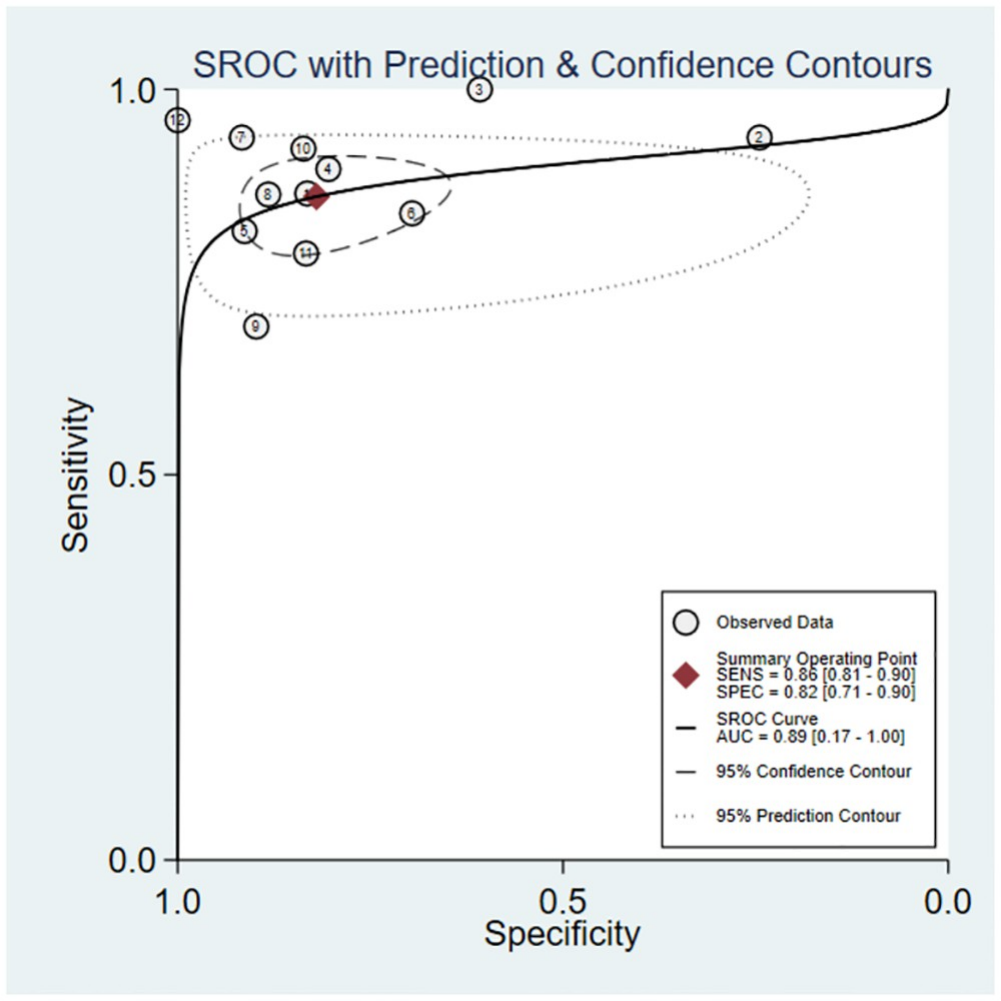
Summary receiver operating characteristic.

**Fig 5:**
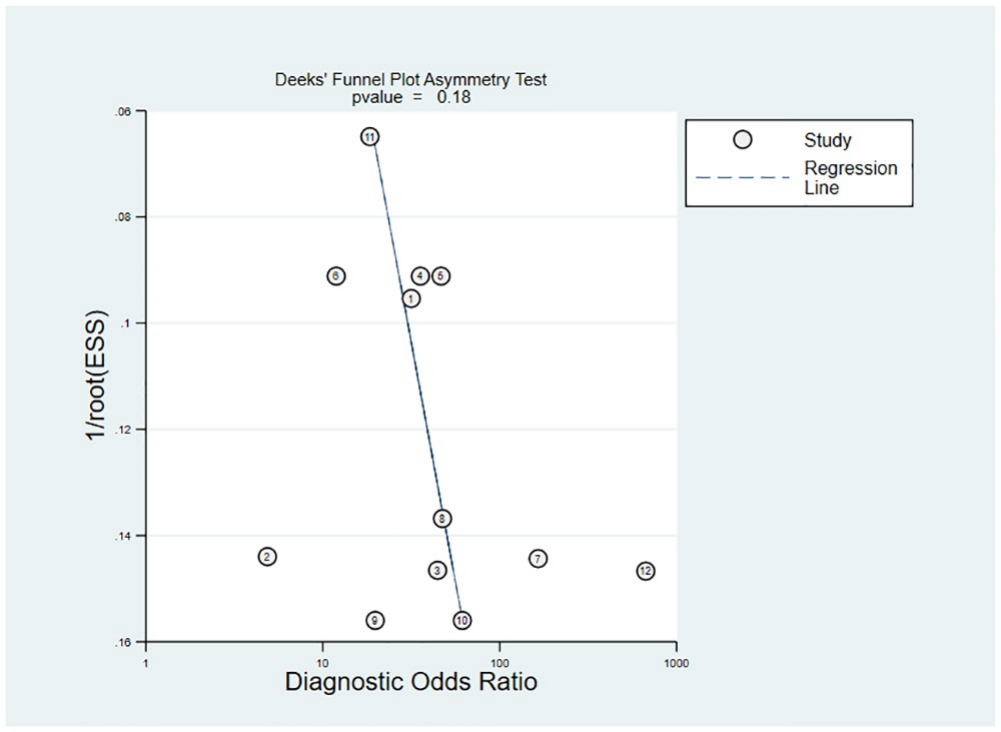
Deeks’ funnel plot.

**Fig 6:**
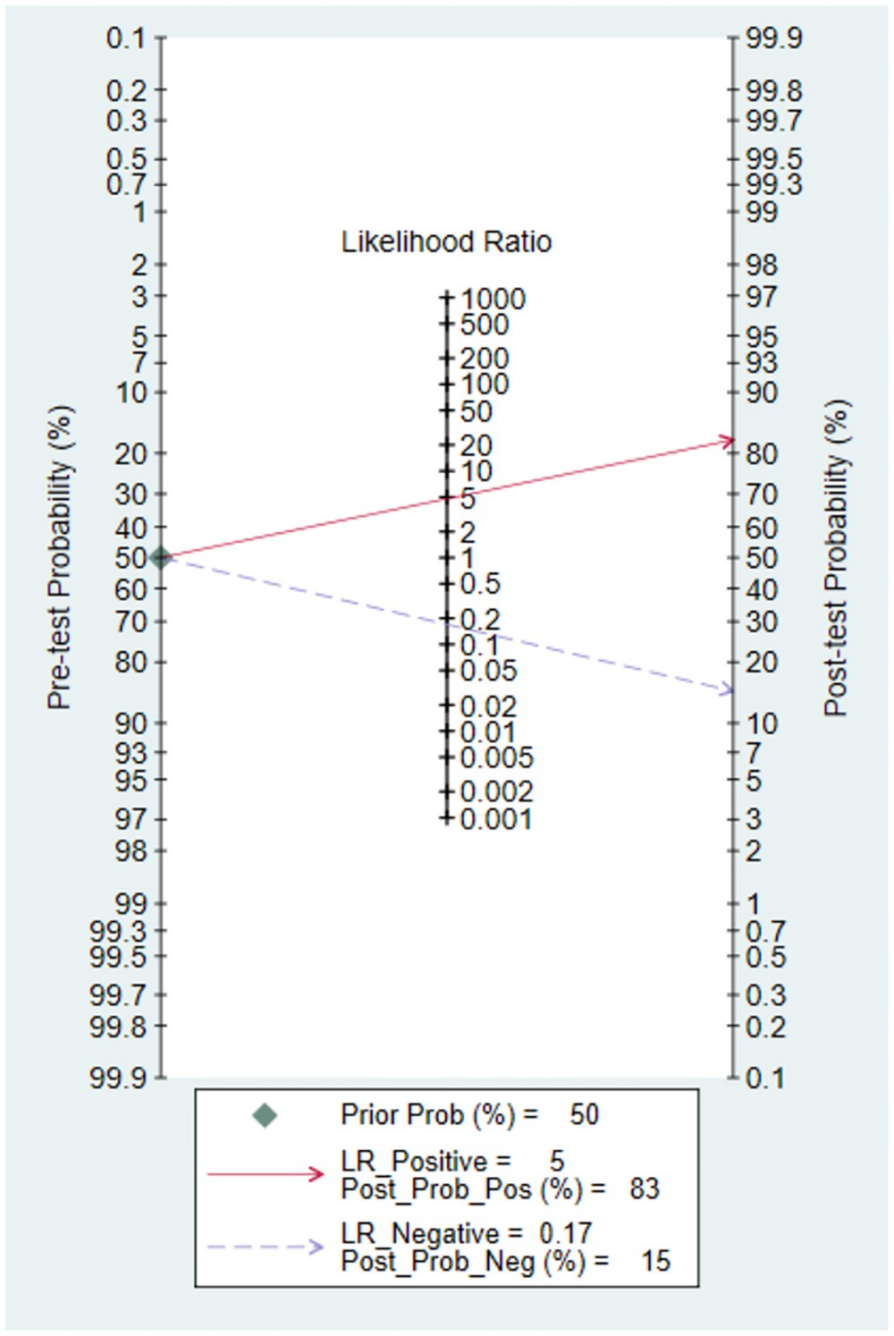
Fagan’s nomogram.

Meta-analysis of the 12 sets of data from 8 studies [9, 15, 17, 18, 20, 22, 23, 26] revealed that lung ultrasound had a sensitivity of 0.86 (95% CI: 0.81–0.90), specificity of 0.82 (95% CI: 0.71–0.90), positive likelihood ratio of 4.8 (95% CI: 2.9–7.9), negative likelihood ratio of 0.17 (95% CI: 0.11–0.23), and SROC of 0.89 (95% CI: 0.17–1.00) in predicting surfactant use. Deeks’ funnel plot showed no publication bias in each study (P = 0.18). Next, we analyzed the clinical applicability of lung ultrasound using Fagan’s nomogram. By assuming a prior probability of 0.5, the posterior probabilities of requiring and not requiring surfactant treatment after lung ultrasound prediction were 0.83 and 0.15, respectively (Figs 3–6).

### Subgroup analysis of timing of lung ultrasound

Since the specific time of lung ultrasound was not provided in the studies by De Martino et al. [17] Raimondi et al. [23] and Vardar et al, [26] we divided the remaining studies into the 1–3 h group and >3 h groups based on the time at which lung ultrasound was performed. The >3 h subgroup was not analyzed due to having only 1 study and 1 set of data [22], which may result in bias. Meta-analysis of the remaining studies showed that lung ultrasound within 1–3 h after birth had a 0.89 (95% CI: 0.79–0.95) sensitivity, 0.78 (95% CI: 0.55–0.91) specificity, and 0.67 Youden’s index for predicting surfactant use (Table 1).

**Table 1:** Subgroup analysis based on timing of lung ultrasound and LUS cutoff.

Subgroup	N	Sensitivity (95%CI)	Specificity (95%CI)	PLR (95%CI)	NLR (95%CI)	DOR (95%CI)	SROC (95%CI)	Youden index
Time								
1~3h	6	0.89[0.79,0.95]	0.78[0.55,0.91]	4.0[1.9,8.5]	0.14[0.07,0.25]	29[12,71]	0.92[0.17–1.00]	0.67
cutoff								
≤5	4	0.94[0.85,0.97]	0.79[0.31,0.97]	4.5[0.8,23.7]	0.08[0.03,0.21]	55[6,525]	0.94[0.30–1.00]	0.73
≤6/7	6	0.92[0.86,0.95]	0.79[0.52,0.93]	4.3[1.6,11.2]	0.11[0.06,0.18]	40[11,147]	0.92[0.37–1.00]	0.71
≤8	8	0.89[0.83,0.93]	0.80[0.63,0.91]	4.5[2.3,8.9]	0.13[0.09,0.20]	34[16,73]	0.92[0.17–1.00]	0.69
>5	8	0.84[0.80,0.87]	0.84[0.80,0.88]	5.3[4.1,6.8]	0.20[0.16,0.24]	27[19,39]	0.89[0.16–1.00]	0.68
>6/7	6	0.82[0.77,0.86]	0.85[0.79,0.90]	5.5[3.8,7.8]	0.21[0.17,0.27]	26[16,42]	0.84[0.15–0.99]	0.67
>8	4	0.81[0.74,0.86]	0.84[0.75,0.91]	5.2[3.2,8.3]	0.23[0.17,0.31]	23[12,42]	0.85[0.32–0.98]	0.65
All	12	0.86[0.81,0.90]	0.82[0.71,0.90]	4.8[2.9,7.9]	0.17[0.11,0.23]	28[15,54]	0.89[0.17–1.00]	0.68

### LUS cutoff

Next, we divided the included studies based on LUS cutoff into the ≤5, ≤6/7, ≤8, > 5, > 6/7, and > 8 groups. Meta-analysis showed that a LUS cutoff of ≤5 had a sensitivity of 0.94 (95% CI: 0.85–0.97), a specificity of 0.79 (95% CI: 0.31–0.97), and the highest Youden’s index of 0.73. (Table 1).

## Discussion

We performed a comprehensive systematic review of studies that investigated the application of lung ultrasound in predicting surfactant use and showed that lung ultrasound is a good predictor for surfactant replacement therapy in neonates with respiratory distress or diagnosed with RDS (sensitivity: 0.86 (95% CI: 0.81–0.90), specificity: 0.82 (95% CI: 0.71–0.90), SROC: 0.89 (95% CI: 0.71–1.00). Subgroup analyses of the timing of lung ultrasound and LUS cutoff revealed that lung ultrasound within 1–3 h of birth and a LUS cutoff of ≤5 had a relatively high sensitivity (0.89 (95% CI: 0.79–0.95) and 0.94 (95% CI: 0.85–0.97), respectively) and Youden’s index (0.67 and 0.73, respectively) for predicting surfactant use.

We determined an optimal LUS cutoff range by compiling the data from each study. As for the timing of lung ultrasound, our results were consistent with those previously reported. The RCT by Rodriguez-Fanjul et al. [25] found that lung ultrasound within 3 h of birth promotes earlier surfactant treatment and reduces oxygen exposure during the early life of neonates. The nested cohort study by Raschetti et al. [24] showed that lung ultrasound-guided surfactant treatment increased the percent of neonates receiving surfactants within 3 h of birth and decreased the maximum FiO2 reached before surfactant replacement therapy. However, due to the small sample size of subgroups, as well as the heterogeneity among studies that resulted in the inability to use lung ultrasound as the absolute indicator for surfactant use, the manifestations and oxygenation of neonatal patients should still be monitored by clinical observation and ventilator parameters, respectively. Perri et al. [22] investigated the use of LUS in the early prediction of CPAP and surfactant, and found that LUS was significantly correlated with SpO2/FiO2 ratio. Raimondi et al. [23] reported that the combination of LUS and SpO2/FiO2 ratio had the highest AUC (0.93 (95% CI: 0.89–0.97)) in predicting surfactant use than each indicator alone.

Based on the results of this meta-analysis, we recommend the expansion of lung ultrasound application to further make use of its noninvasive, radiation-free and bedside advantages such that it is no longer limited to the diagnosis of RDS and TTN but rather involved in the entire diagnosis and treatment process of RDS. To the best of our knowledge, this is the first meta-analysis demonstrating that lung ultrasound is not only a good predictor for surfactant replacement therapy, but lung ultrasound within 1–3 h after birth and a LUS cutoff of 5 also had higher predictive accuracy.

A meta-analysis published in 2020 included 6 studies that evaluated the diagnostic value of LUS in neonates requiring surfactant or mechanical ventilation. The results showed that the risk of surfactant use was higher when LUS is > 5–6 than when LUS is < 5–6, and LUS was more reliable for neonates with a gestational age <34 weeks than for full term and late preterm neonates [31]. However, a shortcoming of this study was that both surfactant use and mechanical ventilation were regarded as one outcome measure when they are clearly different treatment measures. In addition, given the importance of early treatment in RDS, this meta-analysis did not investigate the significance of lung ultrasound timing. Furthermore, several studies published after this meta-analysis indicated that LUS > 5–6 is no longer the optimal cutoff for predicting surfactant use. In contrast, our meta-analysis included several recently published studies, used surfactant as the only primary outcome, and explored in-depth the appropriate timing of lung ultrasound and the optimal LUS cutoff.

Capasso, L. et al. [32] published a meta-analysis on a similar topic to ours. A total of 7 original studies were included in their meta-analysis, which confirmed the predictive value of lung ultrasound for surfactant use. However, in their meta-analysis, there are large differences in the timing of lung ultrasound among the original studies. Further, there are various cut-off thresholds for lung ultrasound scores, but their study did not provide guidance on the cut-off threshold and the timing of lung ultrasound. In clinical practice, for newborns with respiratory distress or diagnosed with RDS, the timing of lung ultrasound and the selection of scoring cut-off thresholds often make front-line medical workers confused. Our meta added 3 newly published original studies on the basis of their meta-analysis, and fully discussed the optimal timing of lung ultrasoundand the cut-off threshold of lung ultrasound score.

Nonetheless, there are still several limitations in this study. First, differences in patient populations, underlying conditions and methods of respiratory support can impact the degree of surfactant deficiency and oxygenation in neonates and increase the heterogeneity among studies. Second, since the small sample size of subgroups, further studies with larger sample size are needed to confirm the results. Finally, most of the included studies used the lung ultrasound diagnostic criteria developed by Brat et al. [9]. Though, there are also several other qualitative and semi-quantitative methods of lung ultrasound assessment. Therefore, more evidence-based studies are warranted to evaluate the significance of these methods.

## Conclusions

Lung ultrasound is a good predictor for surfactant use in neonates with respiratory distress or diagnosed with RDS, and lung ultrasound within 1–3 h after birth and a LUS cutoff of 5 are recommended. Nevertheless, the symptoms and oxygenation of the neonatal patients should still be considered.

## Supporting information

S1 File Detailed search strategy. (PDF)

S1 Table Reasons for article exclusion. (XLSX)

S2 Table Basic characteristics of the included studies. (XLSX)

S1 Checklist PRISMA 2020 checklist. (DOCX)
