# Supplementary material for: Optimal timing and cutoff range of lung ultrasound in predicting surfactant administration in neonates: A meta-analysis and systematic review
Source: PLoS One. 2023 Jul 27;18(7):e0287758. doi: 10.1371/journal.pone.0287758 (PMC10374100; doi:10.1371/journal.pone.0287758)
Supplement: S1 File — (PDF) [file pone.0287758.s001.pdf]

Pubmed		
#	Query	Results
1	"Ultrasounds"[Title/Abstract] OR "Ultrasonographic"[Title/Abstract] OR "Ultrasonic"[Title/Abstract] OR "Echotomography"[Title/Abstract] OR "Diagnostic Ultrasound"[Title/Abstract] OR "Diagnostic Ultrasounds"[Title/Abstract] OR "Ultrasound, Diagnostic"[Title/Abstract] OR "Ultrasounds, Diagnostic"[Title/Abstract] OR "Ultrasound Imaging"[Title/Abstract] OR "Imaging, Ultrasound"[Title/Abstract] OR "Imagings, Ultrasound"[Title/Abstract] OR "Ultrasonic Imaging"[Title/Abstract] OR "Imaging, Ultrasonic"[Title/Abstract] OR "Sonography, Medical"[Title/Abstract] OR "Medical Sonography"[Title/Abstract] OR "Ultrasonographic Imaging"[Title/Abstract] OR "Imaging, Ultrasonographic"[Title/Abstract] OR "Imagings, Ultrasonographic"[Title/Abstract] OR "Ultrasonographic Imagings"[Title/Abstract] OR "Echography"[Title/Abstract] OR "Diagnosis, Ultrasonic"[Title/Abstract] OR "Diagnoses, Ultrasonic"[Title/Abstract] OR "Ultrasonic Diagnoses"[Title/Abstract] OR "Ultrasonic Diagnosis"[Title/Abstract] OR "Echotomography, Computer"[Title/Abstract] OR "Computer Echotomography"[Title/Abstract] OR "Tomography, Ultrasonic"[Title/Abstract] OR "Ultrasonic Tomography"[Title/Abstract]	103982
2	Ultrasonography[MeSH Terms]	477368
3	#1 OR #2	532276
4	"Lungs"[Title/Abstract] OR "Pulmo"[Title/Abstract]	131634
5	"lung"[MeSH Terms]	297484

6	#4 OR #5	370951
7	"infantile respiratory distress syndrome"[Title/Abstract]	14
8	"respiratory distress syndrome, newborn"[MeSH Terms]	15796
9	#7 OR #8	15805
10	"Disease, Hyaline Membrane"[Title/Abstract] OR "Diseases, Hyaline Membrane"[Title/Abstract] OR "Hyaline Membrane Diseases"[Title/Abstract] OR "hyalin membrane disease"[Title/Abstract] OR "hyalin membrane syndrome"[Title/Abstract] OR "hyaline membrane pneumonia"[Title/Abstract] OR "hyaline membrane syndrome"[Title/Abstract] OR "hyalinic membrane disease"[Title/Abstract] OR "neonatal surfactant deficiency"[Title/Abstract] OR "pulmonary hyaline membrane disease"[Title/Abstract] OR "surfactant deficiency disease"[Title/Abstract] OR "surfactant deficiency disorder"[Title/Abstract] OR "surfactant deficiency syndrome"[Title/Abstract] OR "yellow hyaline membrane disease"[Title/Abstract]	140
11	"hyaline membrane disease"[MeSH Terms]	2600
12	#10 OR #11	2638
13	"Transient Tachypnea of Newborn"[Title/Abstract] OR "Newborn Transient Tachypnea"[Title/Abstract] OR "Newborn Transient Tachypneas"[Title/Abstract] OR "neonatal transient tachypnea"[Title/Abstract] OR "neonatal transitory tachypnea"[Title/Abstract] OR "transient tachypnoea of the newborn"[Title/Abstract] OR "transitory tachypnea of newborn"[Title/Abstract] OR "transitory tachypnoea of	130

	newborn"[Title/Abstract]	
14	"transient tachypnea of the newborn"[MeSH Terms]	151
15	#13 OR #14	249
16	#9 OR #12 OR #15	15891
17	#3 AND #6 AND #16	150

Embase		
#	Query	Results
1	'ultrasounds':ti,ab,kw OR 'ultrasonographic':ti,ab,kw OR 'ultrasonic':ti,ab,kw OR 'echotomography':ti,ab,kw OR 'diagnostic ultrasound':ti,ab,kw OR 'diagnostic ultrasounds':ti,ab,kw OR 'ultrasound, diagnostic':ti,ab,kw OR 'ultrasounds, diagnostic':ti,ab,kw OR 'ultrasound imaging':ti,ab,kw OR 'imaging, ultrasound':ti,ab,kw OR 'imagings, ultrasound':ti,ab,kw OR 'ultrasonic imaging':ti,ab,kw OR 'imaging, ultrasonic':ti,ab,kw OR 'sonography, medical':ti,ab,kw OR 'medical sonography':ti,ab,kw OR 'ultrasonographic imaging':ti,ab,kw OR 'imaging, ultrasonographic':ti,ab,kw OR 'imagings, ultrasonographic':ti,ab,kw OR 'ultrasonographic imagings':ti,ab,kw OR 'echography':ti,ab,kw OR 'diagnosis, ultrasonic':ti,ab,kw OR 'diagnoses, ultrasonic':ti,ab,kw OR 'ultrasonic diagnoses':ti,ab,kw OR 'ultrasonic diagnosis':ti,ab,kw OR 'echotomography, computer':ti,ab,kw OR 'computer echotomography':ti,ab,kw OR 'tomography, ultrasonic':ti,ab,kw OR 'ultrasonic tomography':ti,ab,kw	134240
2	'ultrasound'/exp	213710

3	#1 OR #2	309424
4	'lungs':ti,ab,kw OR 'pulmo':ti,ab,kw	174137
5	'lung'/exp	410901
6	#4 OR #5	507435
7	'respiratory distress syndrome, infant':ti,ab,kw	2
8	'neonatal respiratory distress syndrome'/exp	9997
9	#7 OR #8	9999
10	'disease, hyaline membrane':ti,ab,kw OR 'diseases, hyaline membrane':ti,ab,kw OR 'hyaline membrane diseases':ti,ab,kw OR 'hyalin membrane disease':ti,ab,kw OR 'hyalin membrane syndrome':ti,ab,kw OR 'hyaline membrane pneumonia':ti,ab,kw OR 'hyaline membrane syndrome':ti,ab,kw OR 'hyalinic membrane disease':ti,ab,kw OR 'neonatal surfactant deficiency':ti,ab,kw OR 'pulmonary hyaline membrane disease':ti,ab,kw OR 'surfactant deficiency disease':ti,ab,kw OR 'surfactant deficiency disorder':ti,ab,kw OR 'surfactant deficiency syndrome':ti,ab,kw OR 'yellow hyaline membrane disease':ti,ab,kw	179
11	'hyaline membrane disease'/exp	4883
12	#10 OR #11	4937
13	'transient tachypnea of newborn':ti,ab,kw OR 'newborn transient tachypnea':ti,ab,kw OR 'newborn transient tachypneas':ti,ab,kw OR 'neonatal transient tachypnea':ti,ab,kw OR 'neonatal transitory tachypnea':ti,ab,kw OR 'transient tachypnoea of the newborn':ti,ab,kw OR	214

	'transitory tachypnea of newborn':ti,ab,kw OR 'transitory tachypnoea of newborn':ti,ab,kw	
14	'transient tachypnea of the newborn'/exp	875
15	#13 OR #14	978
16	#9 OR #12 OR #15	15260
17	#3 AND #6 AND #16	48

Cochrane Library		
#	Query	Results
1	MeSH descriptor: [Ultrasonography] this term only	5113
2	(“Ultrasounds” or “Ultrasonographic” or “Ultrasonic” or “Echotomography” or “Diagnostic Ultrasound” or “Diagnostic Ultrasounds” or “Ultrasound, Diagnostic” or “Ultrasounds, Diagnostic” or “Ultrasound Imaging” or “Imaging, Ultrasound” or “Imagings, Ultrasound” or “Ultrasonic Imaging” or “Imaging, Ultrasonic” or “Sonography, Medical” or “Medical Sonography” or “Ultrasonographic Imaging” or “Imaging, Ultrasonographic” or “Imagings, Ultrasonographic” or “Ultrasonographic Imagings” or “Echography” or “Diagnosis, Ultrasonic” or “Diagnoses, Ultrasonic” or “Ultrasonic Diagnoses” or “Ultrasonic Diagnosis” or “Echotomography, Computer” or “Computer Echotomography” or “Tomography, Ultrasonic” or “Ultrasonic Tomography”):ti,ab,kw	42391

3	#1 OR #2	44363
4	MeSH descriptor: [Lung] this term only	3478
5	(“Lungs” or “Pulmo”):ti,ab,kw	79972
6	#4 OR #5	79972
7	MeSH descriptor: [Respiratory Distress Syndrome, Newborn] this term only	1685
8	(Infantile Respiratory Distress Syndrome):ti,ab,kw	30
9	#7 OR #8	1709
10	MeSH descriptor: [Hyaline Membrane Disease] this term only	100
11	(“Disease, Hyaline Membrane” or “Diseases, Hyaline Membrane” or “Hyaline Membrane Diseases” or “hyalin membrane disease” or “hyalin membrane syndrome” or “hyaline membrane pneumonia” or “hyaline membrane syndrome” or “hyalinic membrane disease” or “neonatal surfactant deficiency” or “pulmonary hyaline membrane disease” or “surfactant deficiency disease” or “surfactant deficiency disorder” or “surfactant deficiency syndrome” or “yellow hyaline membrane disease”):ti,ab,kw	188
12	#10 OR #11	188
13	MeSH descriptor: [Transient Tachypnea of the Newborn] this term only	47
14	(“Transient Tachypnea of Newborn” or “Newborn Transient Tachypnea” or “Newborn Transient Tachypneas” or “neonatal transient tachypnea” or “neonatal transitory tachypnea” or “transient	167

	tachypnoea of the newborn" or "transitory tachypnea of newborn" or "transitory tachypnoea of newborn"):ti,ab,kw	
15	#13 OR #14	167
16	#9 OR #12 OR #15	2006
17	#3 AND #6 AND #16	33

Web of Science		
#	Query	Results
1	Ultrasonography (Topic) OR Ultrasound (Topic) OR Ultrasounds (Topic) OR Ultrasonographic (Topic) OR Ultrasonic (Topic) OR Echotomography (Topic) OR Diagnostic Ultrasound Topic) OR Diagnostic Ultrasounds (Topic) OR Ultrasound, Diagnostic (Topic) OR Ultrasounds, Diagnostic (Topic) OR Ultrasound Imaging (Topic) OR Imaging, Ultrasound (Topic) OR Imagings, Ultrasound (Topic OR Ultrasonic Imaging (Topic) OR Imaging, Ultrasonic (Topic) OR Sonography, Medical (Topic) OR Medical Sonography (Topic) OR Ultrasonographic Imaging (Topic) OR Imaging, Ultrasonographic (Topic) OR Imagings, Ultrasonographic (Topic) OR Ultrasonographic Imagings (Topic) OR Echography (Topic) OR Diagnosis, Ultrasonic (Topic) OR Diagnoses, Ultrasonic (Topic) OR Ultrasonic Diagnoses (Topic) OR Ultrasonic Diagnosis (Topic) OR Echotomography, Computer (Topic) OR Computer Echotomography (Topic) OR Tomography, Ultrasonic (Topic) OR Ultrasonic Tomography (Topic)	621520
2	Lung (Topic) or Lungs (Topic) or Pulmo (Topic)	907240
3	Respiratory Distress Syndrome, Newborn (Topic) or Infantile Respiratory	12856

	Distress Syndrome (Topic) or Neonatal Respiratory Distress Syndrome (Topic) or Respiratory Distress Syndrome, Infant (Topic) or Hyaline Membrane Disease (Topic) or Disease, Hyaline Membrane (Topic) or Diseases, Hyaline Membrane (Topic) or Hyaline Membrane Diseases (Topic) or hyalin membrane disease (Topic) or hyalin membrane syndrome (Topic) or hyaline membrane pneumonia (Topic) or hyaline membrane syndrome (Topic) or hyalinic membrane disease (Topic) or neonatal surfactant deficiency (Topic) or pulmonary hyaline membrane disease (Topic) or surfactant deficiency disease (Topic) or surfactant deficiency disorder (Topic) or surfactant deficiency syndrome (Topic) or yellow hyaline membrane disease (Topic) or Transient Tachypnea of the Newborn (Topic) or Transient Tachypnea of Newborn (Topic) or Newborn Transient Tachypnea (Topic) or Newborn Transient Tachypneas (Topic) or neonatal transient tachypnea (Topic) or neonatal transitory tachypnea (Topic) or transient tachypnoea of the newborn (Topic) or transitory tachypnea of newborn (Topic) or transitory tachypnoea of newborn (Topic)	
4	#1 AND #2 AND #3	287
